# Increased plasma ANGPTL7 levels with increased obstructive sleep apnea severity

**DOI:** 10.3389/fendo.2022.922425

**Published:** 2022-08-09

**Authors:** M. Leentjens, Abdulmohsen Alterki, Mohamed Abu-Farha, P. F. N. Bosschieter, CAL. de Raaff, CEE. de Vries, Eman Al Shawaf, Thangavel Alphonse Thanaraj, Irina Al-Khairi, Preethi Cherian, Arshad Channanath, Sina Kavalakatt, B. A. van Wagensveld, N. de Vries, Jehad Abubaker

**Affiliations:** ^1^ Department of Otorhinolaryngology - Head and Neck Surgery, Onze Lieve Vrouwe Gasthuis (OLVG) Hospital, Amsterdam, Netherlands; ^2^ Department of Otolaryngology - Head and Neck Surgery, Zain and Al Sabah Hospitals and Dasman Diabetes Institute, Kuwait City, Kuwait; ^3^ Department of Biochemistry and Molecular Biology, Dasman Diabetes Institute, Kuwait City, Kuwait; ^4^ Department of Surgery, Amsterdam University Medical Center (UMC), Amsterdam, Netherlands; ^5^ Department of Surgery, Reinier de Graaf Gasthuis, Delft, Netherlands; ^6^ Department of Genetics and Bioinformatics, Dasman Diabetes Institute, Dasman, Kuwait; ^7^ Obesity Department, New Medical Centre (NMC) Royal Hospital Khalifa City, Abu Dhabi, United Arab Emirates; ^8^ Department of Oral Kinesiology, Academic Centre for Dentistry in Amsterdam (ACTA), Move Research Institute Amsterdam, University of Amsterdam and Vrije University (VU) University Amsterdam, Amsterdam, Netherlands; ^9^ Department of Otorhinolaryngology - Head and Neck Surgery, Faculty of Medicine and Health Sciences, Antwerp University Hospital, Antwerp, Belgium

**Keywords:** obstructive sleep apnea, ANGPTL7, hypoxia, polysomnography, apnea hypopnea index, bariatric surgery

## Abstract

**Background:**

Weight-loss surgery is one of the recommended methods for treating obstructive sleep apnea (OSA) in obese patients. While weight reduction is critical to relieve symptoms of OSA, the biochemical factors involved in post-surgery improvement are still unknown. We aimed to explore the link between ANGPTL7 and OSA in patients with different OSA severity. Furthermore, we examined the effect of treating OSA with bariatric surgery on ANGPTL7 level.

**Methods:**

We quantified levels of circulating ANGPTL7 in fasting plasma and adipose tissue samples of 88 participants before and after bariatric surgery. Confocal microscopy analyses were also performed to assess the ANGPTL7 expression in subcutaneous white adipose tissue biopsies obtained from people with moderate-to-severe OSA compared to those with none or mild OSA. The study involved 57 individuals with none or mild OSA and 31 patients with moderate-to-severe OSA.

**Results:**

Levels of circulating ANGPTL7 were significantly higher in people with moderate-to-severe OSA (1440 ± 1310 pg/ml) compared to the none or mild OSA group (734 ± 904 pg/ml, *p* = 0.01). The increase in ANGPTL7 correlated significantly and positively with the apnea-hypopnea index (AHI, r = .226, *p* = .037), and AHI-supine (r = .266, *p* = .019) in participants with moderate-to-severe OSA. Multivariate logistic regression analysis demonstrated an association between ANGPTL7 and OSA severity (log_2_ ANGPTL7; OR =1.24, *p* = 0.024). ANGPTL7 levels exhibited significant positive correlations with the levels of TG and oxLDL (p-value = 0.002 and 0.01 respectively). Bariatric surgery reduced the levels of both ANGPTL7 and AHI significantly.

**Conclusion:**

Here we report significantly increased levels of ANGPTL7 both in the circulation and in adipose tissue of patients with OSA, which concurred with increased inflammation and OSA severity. Levels of ANGPTL7 decreased significantly as OSA showed a significant improvement post-surgery supporting a potential role for ANGPTL7 in either OSA progression or a role in an OSA-related mechanism.

## Introduction

Obstructive sleep Apnea (OSA) is a chronic sleep disorder that is common in people with obesity. It is a condition of fragmented sleep that is accompanied by repeated episodes of disrupted air flow, apnea or hypopnea, which results in intermittent hypoxia, oxyhemoglobin desaturation, interrupted sleep and daytime sleepiness (1). The chronic presence of airflow disruptions triggers pathological mechanisms that might evoke vascular damage leading to the development of cardiovascular morbidities (1, 2). Oxidative stress with a state of systemic inflammation, the expression and release of adhesion molecules and endothelial dysfunction are recognized consequences of OSA (3). Thus, having OSA is an independent predisposing factor for vascular dysfunction and its consequences. Levels of several inflammatory markers were found to increase with OSA severity, and they served as predictive indicators of potential OSA comorbidities. C-reactive protein (CRP) is an acute-phase protein that has emerged as a strong predictor of cardiovascular risk, and levels of CRP were elevated with increased OSA severity (4). Interleukin-6 (IL-6) is a proinflammatory cytokine that was found to increase with sleep deprivation and intermittent hypoxia and its levels were also higher with increased OSA severity (5, 6).

OSA treatment options involve lifestyle modifications, weight reduction, continuous positive airway pressure (CPAP), mandibular advancement devices (MAD), positional therapy in position dependent OSA, and use of surgical procedures or hypoglossal nerve stimulation. Studies found weight loss to concur with a reduced apnea-hypopnea index (AHI) and to improve apnea symptoms in patients with obesity and OSA (7, 8). Due to the rapid and prolonged benefits of weight-loss surgeries, clinical guidelines for bariatric surgery strongly recommends considering a surgical weight-loss procedure to treat patients with morbid obesity and OSA (9). Although loss of excess weight per se is an important factor to relieve symptoms of OSA, and reduce its severity or even cure it, there are still unknown mechanisms and biochemical factors associated with obesity and OSA that are involved in the rapid improvement after bariatric surgery.

A previous study examined angiopoietin-like protein 7 (ANGPTL7) levels in obesity and the effect of physical activity on its expression level (10). This protein is a member of the ANGPTL family, which comprises eight different proteins that were found to have autocrine and paracrine activities and were associated with inflammation and angiogenesis (11, 12). Although ANGPTL7 is one of the least explored ANGPTLs, there is a growing interest to decipher its potential and physiological role. Previous reports demonstrated the role of ANGPTL7 in inflammatory responses, which are involved in the pathogenesis of heart failure. ANGPTL7 was shown to regulate expression of genes encoding Tumor necrosis factor alpha (TNF-α), interleukin-1 beta (IL-1β), IL-6, inducible nitric oxide synthase (iNOS) and cyclooxygenase-2 (COX-2) (13). Another study reported increased ANGPTL7 expression in people with hypertension that alluded to a role in vascular remodeling (14). In a recent study, Xu and colleagues demonstrated ANGPTL7 as a potential therapeutic target for the treatment of insulin resistance and type 2 diabetes (T2D) (15). ANGPTL7 was found to play a critical role in inducing insulin resistance, which occurred through multiple mechanisms involving the down regulation of Insulin receptor (INSR), upregulation of suppressor of cytokine signaling 3 protein (SOCS3) to degrade insulin receptor substrate 1 (IRS1) (15). Because of its role in inflammation, insulin resistance and T2D we hypothesized that ANGPTL7 expression level might be affected by OSA.

This study aimed to examine the effect of bariatric surgery on ANGPTL7 levels and to compare these levels to pre- and postoperative polygraphy (PG) or polysomnography (PSG) outcomes. Thus, the involvement of and the correlation between ANGPTL7 and OSA in a Dutch cohort of patients with morbid obesity were explored.

## Materials and methods

### Study design and ethical statement

This is a prospective cohort study involving patients that were scheduled for primary Laparoscopic Sleeve Gastrectomy (LSG) or Laparoscopic Roux-en-Y gastric bypass (LRYGB) in the OLVG Hospital, Amsterdam, the Netherlands. Exclusion criteria were medical history of cardiovascular disease (CVD) or any major chronic diseases and patients needing revisional surgery. The study was conducted in accordance with the ethical guidelines outlined in the Declaration of Helsinki and it was approved by the medical ethical committee. All patients gave written informed consent prior to their enrollment in the study.

### Data collection

Enrolled patients were entered in a database. Baseline characteristics and PG or PSG outcomes were collected from their medical records. Patients were divided into two groups according to AHI with a cutoff point of fifteen events/hour: none or mild OSA (AHI 0-15 events/hour) and moderate-to-severe OSA (AHI ≥15 events/hour). Patients with moderate-to-severe OSA were referred to the pulmonologist for treatment with continuous positive airway pressure (CPAP). Adipose tissue samples were collected from all patients at time of surgery. All patients were invited for a PG or PSG six to nine months after surgery. Plasma collection from venipuncture was obtained prior to surgery and six to nine months after surgery.

### Anthropometry and biochemical measurement

Fasting blood samples were collected from all patients before and after surgery. Fasting blood glucose (FBG), serum total cholesterol (TC), low-density lipoprotein (LDL), high-density lipoprotein (HDL) and triglycerides (TG) were measured using Siemens Dimension RXL chemical analyzer (Diamond Diagnostics, Holliston, MA, USA). Glycated hemoglobin (HbA1c) was quantified using VariantTM device (Bio-Rad, Hercules, CA). Also, samples were collected in vacutainer EDTA tubes and plasma was separated by 10 min centrifugation at 400 X g. Collected plasma was aliquoted and stored at −80 °C until used in further analysis. The collected adipose tissue biopsies (~5 g) were processed and stored appropriately until assayed. The samples were packed in dry ice and then send to Dasman Diabetes Institute (DDI) in Kuwait City, Kuwait for further assessment.

### Quantification of metabolites and biomarkers

Protein specific Enzyme-linked immunosorbent assays (ELISA) assays were performed to quantify plasma biomarkers. Levels of both the insulin and c-peptide were measured to calculate insulin resistance and the level of endogenous insulin production. Plasma levels of metabolic markers were determined using multiplexing immunobead array platform (Luminex, Austin, TX). The Human Diabetes 10-Plex and the inflammation related-27plex cytokine kits were used to cover a wide range of metabolic markers related to OSA. ox-LDL concentrations were determined using ox-LDL/MDA Addukt ELISA kit (K7810) – Immunodiagnostik). All measurements followed the manufacturers’ instructions. Identification of biomarkers involved in OSA was achieved using the same method as described in a previous study (16), by following the approach of quantitative targeted metabolic analysis.

### Measurement of gene expression by real-time quantitative PCR

Total RNA was extracted from frozen adipose tissue using RNeasy Lipid Tissue Mini Kit (Qiagen, Inc., Valencia, CA). Total RNA was isolated from adipose tissue biopsies. The cDNA was prepared from total RNA sample using High Capacity cDNA Reverse Transcription Kits (Applied Biosystems, Foster City, CA). The gene expression analysis for ANGPTL7 was performed using the same method as in a previous study (10). In brief, qRT-PCR was performed on Rotor-Disc 100 system using SYBR Green normalized to Gapdh (Qiagen, Inc., Valencia, CA). PCR primers used were: ANGPTL7 For., 5’-TAGAGATGGAGGACTG-GGAGG-3’; ANGPTL7 Rev., 5’- GTGCACACTTGCCAAGCAG-3’; Gapdh For., 5′- AACTTTGGCATTGTGGAAGG-3’ and Gapdh Rev., 5′-TGTGAGGG-AGATGCTCAGTG-3’. Relative expression was assessed by using the ΔΔCT method (15).

### Immunofluorescence and confocal microscopy

For confocal imaging, formaldehyde-fixed subcutaneous adipose tissue was embedded in paraffin and made into sections with a thickness of eight μm. The prepared tissue sections were deparaffinized, rehydrated and subjected to antigen retrieval using DAKO reagents (Dako, Glostrup, Denmark). Tissue sections were incubated in 3% hydrogen peroxide for one hour at room temperature (RT) to quench endogenous peroxidase activity, followed by sequential blocking with 5% fat free milk and 1% bovine serum albumin for one hour at room temperature. Following antigen retrieval and blocking, tissue sections were incubated with the ANGPTL7 primary antibody (LS-C119727, Lifespan Biosciences, USA) overnight at 4°C. To assess antibody specificity, we performed negative controls by incubating sections without the primary antibody. This was followed by incubating sections with secondary antibody Alexa fluor 488 conjugated antibody (A-11008, Invitrogen, USA) at 1:1000 dilution for one hour at room temperature. For nuclear staining and mounting sections, we used mounting media with DAPI (H-1200, Vector laboratories, USA). Imaging of samples was performed using Zeiss LSM 710 confocal laser scanning microscope (Zeiss, Germany). Fluorescent images were captured at 40 x objective, and image intensities were quantified with the Zen software (Zeiss, Germany).

### Statistical analysis

Data was reported as mean (± standard deviation) if normally distributed and median (IQR) if not normally distributed. Categorical variables were expressed as absolute values and percentages. Spearman’s correlation tests were performed to study the relationship between ANGPTL7 and other clinical parameters. Comparing the levels of biochemical parameters between none or mild OSA (AHI <15 events/hour) and moderate-to-severe OSA (AHI ≥ 15 events/hour) was performed using unpaired t-test in case of normally distributed data or Mann-Whitney U-test if not normally distributed. Paired t-tests were used to determine the statistical significance of mean differences in the levels of biochemical parameters before and after surgery, in case of not normally distributed data a Wilcoxon signed-rank test was performed. Differences in the categorical variables between the groups were analyzed using chi-squared (χ2) tests. To evaluate the association between ANGPTL7 and OSA severity, multivariate binary logistic regression was performed. The none or mild OSA was set as the reference group. Covariates, age and gender, for the multivariate regression was enrolled based on the statistical significance level (p < 0.1) of the variable in univariate logistic regression models. A p-value < 0.05 was considered as statistically significant. All statistical analyses were performed using R statistical software (R Core Team, 2020).

## Results

### Baseline characteristics

**Figure 1:**
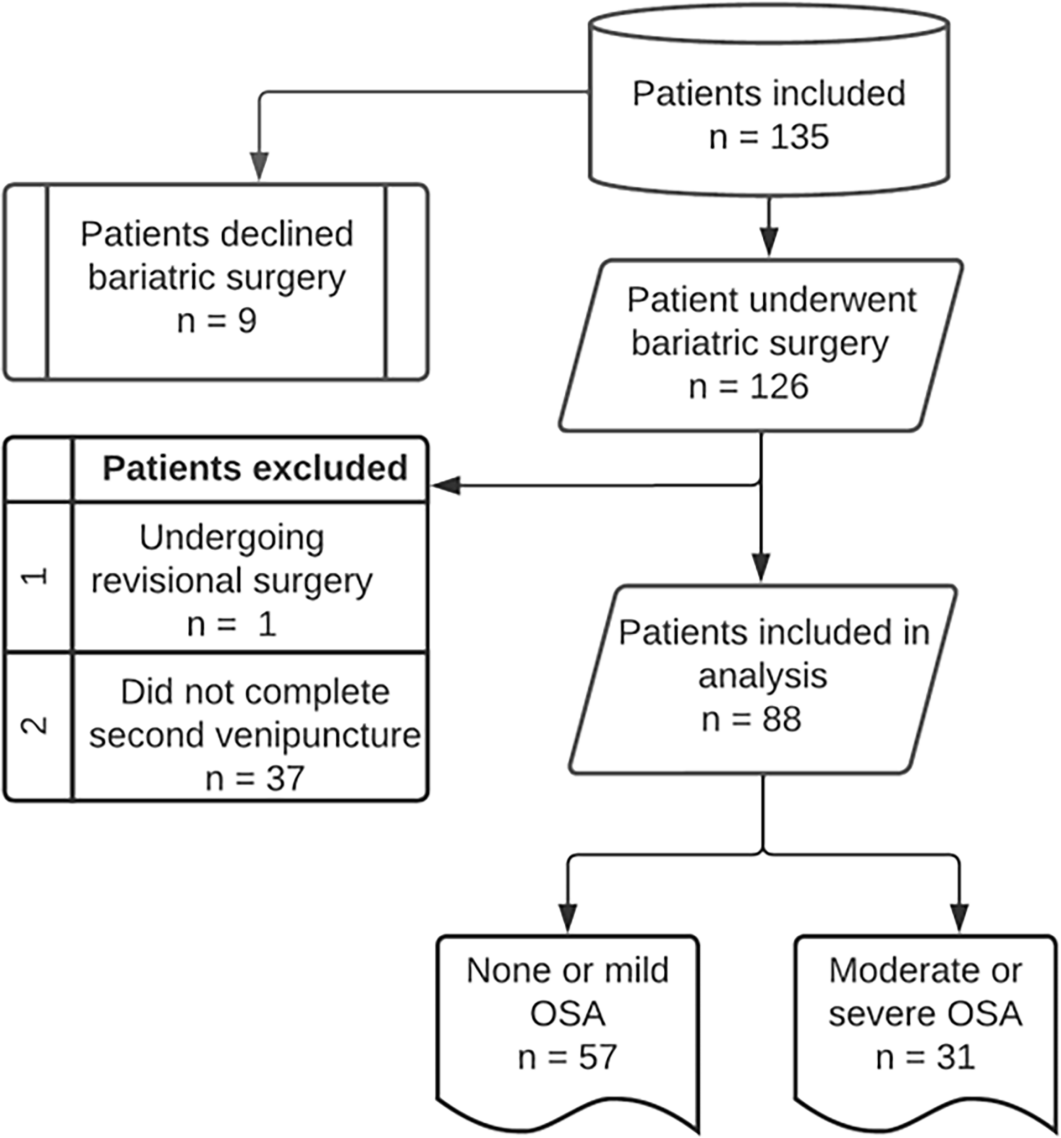
Flowchart of subjects included in the study.

**Figure 2:**
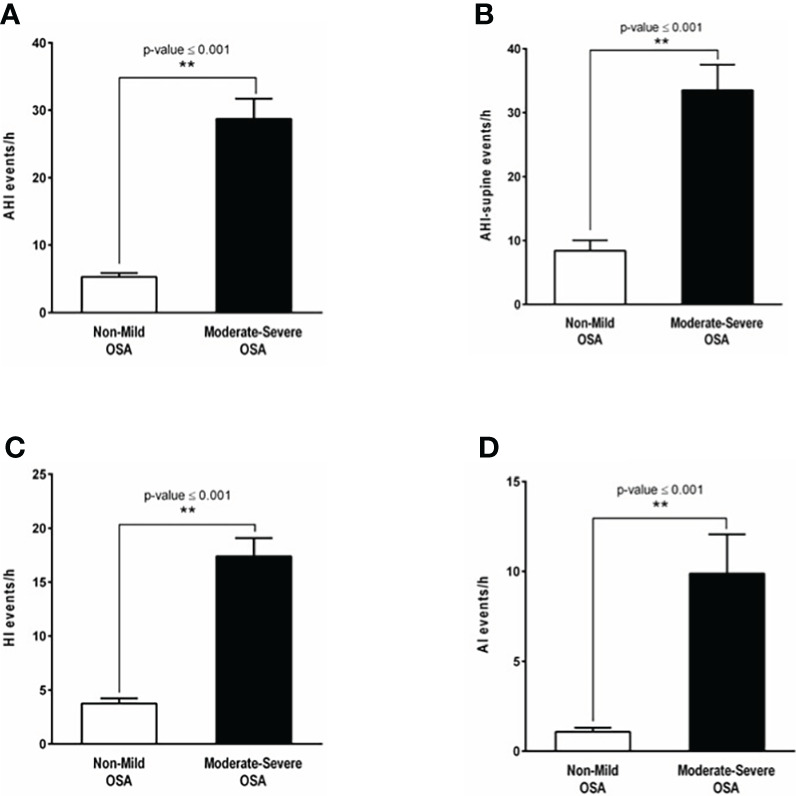
OSA diagnosis by polysomnography and OSA indices. **(A)** The apnea hypopnea index (AHI) demonstrating a significant difference in OSA severity between patients with none or mild OSA (5.27 ± 0.57 events/hour, white bar) and those with moderate-to-severe OSA (28.7 ± 3.02 events/hour, p-value <0.001, black bar). **(B)** Apnea hypopnea-supine index (AHI-supine) reflecting a significant increase in OSA severity in patients with moderate-to-severe OSA (33.5 ± 4 events/hour, p-value <0.001, black bar) compared to the none or mild OSA group (8.41 ± 1.63 events/hour, white bar). **(C)** The number of shallow breathing events reflected by the hypopnea index (HI) showing a significant increase in patients with moderate-to-severe OSA (17.4 ± 9.29 events/hour, p-value <0.001, black bar) in comparison to those with none or mild OSA (3.76 ± 3.55 events/hour, white bar). **(D)** The apnea index (AI) showing that the number of complete paused breathing events in patients with moderate-to-severe OSA (9.88 ± 12 events/hour, p-value <0.001, black bar) is significantly higher compared to patients with none or mild OSA (1.08 ± 1.74 events/hour, white bar). **: p-value <0.001.

A total of 135 patients were included in this prospective cohort study. Nine patients declined bariatric surgery. Thus, 126 patients underwent a weight loss surgical intervention between 2016 and 2018. One patient was excluded due to revisional surgery, 37 patients did not complete all follow-up measurements, such as venipuncture and post-surgery PG or PSG. Therefore, a total of 88 patients were included for analysis (**Figure 1**). These patients were subdivided in a none or mild OSA group (n=57) and a moderate-to-severe OSA group (n=31). The majority of patients were females, 94% in the none or mild OSA group and 80% in the moderate-to-severe OSA group (p-value = 0.086). There was a significant difference in the age (50.6 ± 11.1 vs 44.4 ± 12.2 years; p-value =0.029) of patients with moderate-to-severe OSA compared to those with none or mild OSA. Polysomnography data for both the none-to-mild OSA group as well as the moderate-to-severe OSA group is shown in (**Figure 2**), with Apnea-hypopnea index (AHI =28.7 ± 3.02 events/h) in moderate-to-severe OSA compared with the none or mild OSA group (5.27 ± 0.57 events/h, **Figure 2A**). Additionally, body position during sleep appears to have a substantial effect on the severity of sleep-related breathing disturbances, as reflected by the observed significant increase in AHI-supine (**Figure 2B**) in patients with moderate-to-severe OSA (33.5 ± 4 events/h, p-value <0.001) compared to the none or mild OSA group (8.41 ± 1.63 events/h). Both AI and HI showed significant differences between the two groups in OSA severity. Patients with moderate-to-severe OSA experienced higher number of apneic events (9.88 ± 12 events/h) compared to the none or mild OSA group (1.08 ± 1.74 events/h, **Figure 2C**). Similarly, patients with moderate-to-severe OSA had significantly high number of hypopneic events (17.4 ± 9.29 events/h, p-value <0.001) compared to the none or mild OSA group (3.76 ± 3.55 events/h, **Figure 2D**).

There was a significant difference in TG (2.30 ± 0.998 mmol/L vs. 1.83 ± 0.853 mmol/L; p-value = 0.03) in patients with moderate-to-severe OSA compared to those with none or mild OSA. An increase in inflammatory markers (such as IL-6, IL-10 and TNFα) was seen in patients with moderate-to-severe OSA compared to patients with none or mild OSA, but with no statistical significance (**Table 1**).

**Table 1:** Comparison of baseline characteristics and measurements.

	None or mild OSA (AHI < 15 events/hour)	Moderate-to-Severe OSA (AHI ≥ 15 events/hour)	P-value
	(N=57)	(N=31)	
**Gender**			
Female	54 (94.7%)	25 (80.6%)	0.086
Male	3 (5.3%)	6 (19.4%)	
**Age (years)**	44.4 ± 12.2	50.6 ± 11.1	0.029
**BMI (kg/m^2^)**	41.9 ± 5.64	43.7 ± 8.84	0.71
**c-peptide (pg/ml)**	5750 ± 2170	5780 ± 2250	0.702
**Insulin (U/L)**	19.8 (9.3, 51.0)	21.0 (9.8, 48.5)	0.829
**Glucose (mmol/L)**	5.6 (5.0, 6.9)	6.7 (5.3, 8.4)	0.134
**AHI (events/h)**	5.27 ± 4.30	28.7 ± 16.8	**<0.001**
**AI (events/h)**	1.08 ± 1.74	9.88 ± 12.0	**<0.001**
**HI (events/h)**	3.76 ± 3.55	17.4 ± 9.29	**<0.001**
**AHI-supine (events/h)**	8.41 ± 11.9	33.5 ± 21.2	**<0.001**
**CPAP (cm H_2_O)**	0.0175 ± 0.132	0.968 ± 0.180	<0.001
**Cholesterol (mmol/L)**	5.26 ± 0.987	5.14 ± 0.879	0.647
**Triglyceride (mmol/L)**	1.83 ± 0.853	2.30 ± 0.998	**0.03**
**HDL (mmol/L)**	1.30 ± 0.330	1.24 ± 0.352	0.14
**LDL (mmol/L)**	3.40 ± 0.949	3.27 ± 0.788	0.567
**ox-LDL(ng/ml)**	48.3 (32.3, 125.5)	60.7 (35.4, 95.3)	0.6
**ANGPTL7 (pg/ml)**	435.0 (65.0, 1,135.0)	1,170.0 (322.5, 2,585.0)	**0.02**
**IL_6 (ng/ml)**	1.4 (0.0, 2.6)	1.8 (0.8, 2.5)	0.379
**IL_10 (ng/ml)**	2.1 (1.4, 2.6)	2.1 (1.4, 2.5)	0.782
**TNFα (ng/ml)**	2.0 (0.5, 3.8)	2.6 (1.3, 4.1)	0.2
**Leptin (pg/ml)**	57000 ± 37300	51500 ± 42300	0.213

BMI, Body Mass index; AHI, Apnea Hypopnea Index; AI, Apnea Index; HI, Hypopnea index; HDL, High Density Lipoprotein; LDL, Low Density Lipoprotein; ANGPTL7, Angiopoietin-like protein 7. Significant P-values are indicated in bold font.

### Levels of ANGPTL7 in circulation and adipose tissue

**Figure 3:**
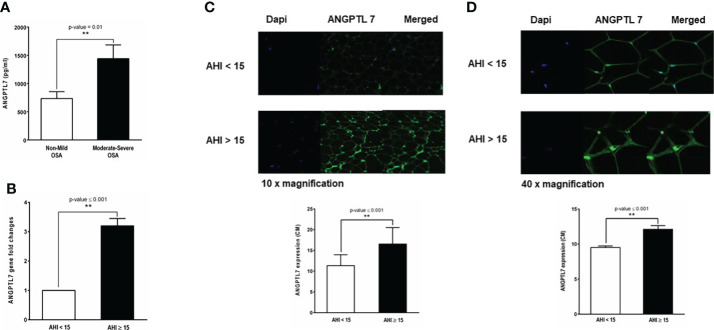
Baseline levels of ANGPTL7 in patients with and without OSA. **(A)** Circulating ANGPTL7 is significantly higher in patients with moderate-to-severe OSA (1440 ±243.46 pg/ml, p-value = 0.01) compared to those with none or mild OSA (734.904 ± 121.85 pg/ml). **(B)** ANGPTL7 gene expression showing 3.2-fold increase in patients with moderate-to-severe OSA compared to patients with none or mild OSA. **(C)** Increased ANGPTL7 protein expression in adipose tissue determined by confocal microscopy. Representative images taken at 10x showing expression level in patients with AHI < 15 events/hour and moderate-to-severe OSA with AHI ≥ 15 events/hour, quantification of ANGPTL7 protein expression plotted as bar chart showing a significant difference in ANGPTL7 protein expression. **(D)** Images of ANGPTL7 protein expression at 40x with a quantification of expression level by a bar chart plot. **: p-value <0.001.

Plasma ANGPTL7 was significantly higher (1440 ± 243.46 pg/ml, p-value= 0.01) in patients with moderate-to-severe OSA compared to the none or mild OSA group (734 ± 121.85 pg/ml, **Figure 3A**). Additionally, there was a significant increase in the expression of ANGPTL7 in subcutaneous white adipose tissue biopsies obtained from patients with moderate-to-severe OSA compared to those with none or mild OSA as pointed out by gene expression and confocal microscopy analyses (**Figures 3B–D**).

### ANGPTL7 correlations

**Figure 4:**
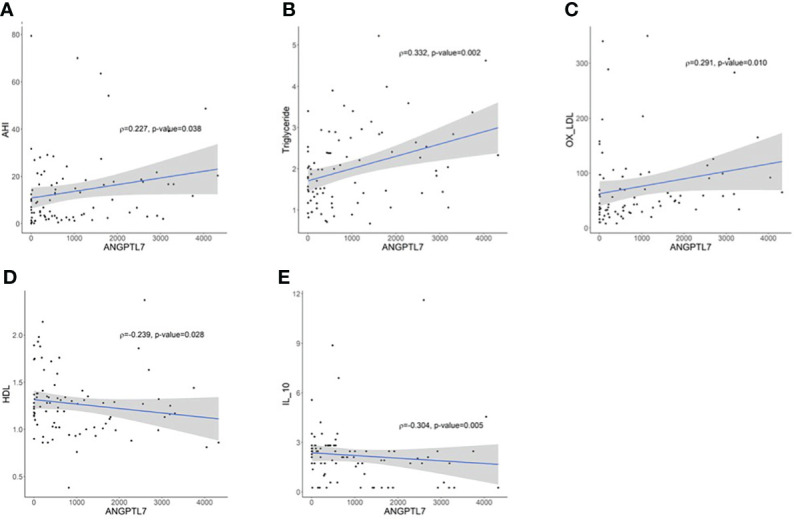
Spearman correlation analysis between circulating ANGPTL7 and; **(A)** apnea-hypopnea index (AHI) that demonstrates a significant positive correlation (ρ= 0.227; p-value= 0.038), **(B)** TG displaying a significant positive correlation (ρ=0.331; p-value=0.002), **(C)** oxLDL reflecting a significant positive correlation (ρ=0.291; p-value=0.010). ANGPTL7 showed a significant negative correlation with **(D)** HDL (ρ=-0.239; p-value=0.028), **(E)** IL-10 (ρ=-0.304; p-value=0.005).

Spearman’s correlation analysis pointed out significant positive correlations between ANGPTL7 and AHI (ρ= 0.227; p-value= 0.038, **Figure 4A**), TG (ρ=0.332; p-value=0.002, **Figure 4B**) and oxLDL (ρ=0.291; p-value=0.010, **Figure 4C**). ANGPTL7 showed a significant negative correlation with HDL (ρ= −0.239; p-value= 0.028, **Figure 4D**) and IL-10 (ρ= −0.303; p-value= 0.005, **Figure 4E**). The positive correlation between OSA indices and ANGPTL7 expression was further corroborated through multivariate logistic regression, which showed an association between ANGPTL7 and OSA severity (log_2_ ANGPTL7; OR = 1.24, 95% CI [1.07, 1.51], p-value = 0.024).

### Bariatric surgery, OSA severity and ANGPTL7 levels

**Figure 5:**
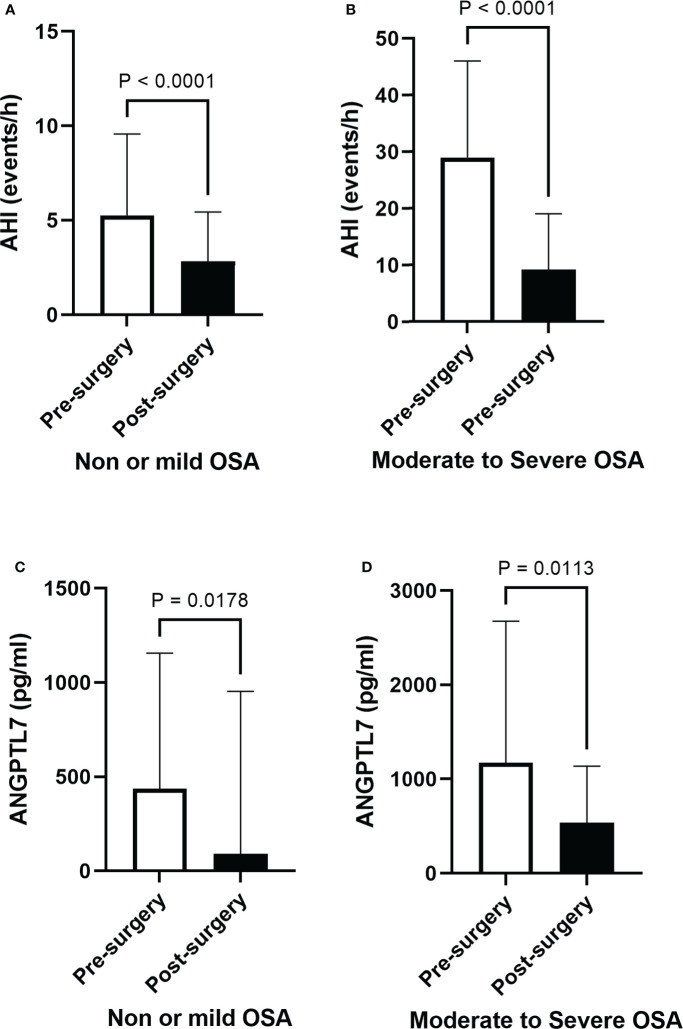
Post-surgery improvement in OSA and a decline in circulating ANGPTL7. Apnea-hypopnea index (AHI) demonstrates a significant decline after six months of bariatric surgery in **(A)** people with none or mild OSA (2.82 ± 2.6 events/hour, p < 0.001) and those with **(B)** moderate-to-severe OSA (9.10 ± 9.69 events/hour, p < 0.001), reflecting a substantial improvement in OSA. Levels of circulating ANGPTL7 dropped in patients with none or mild OSA (114 ± 807 pg/ml, p = 0.014, **(C)**, and it showed a significant reduction in levels of circulating ANGPTL7 in patients with moderate-to-severe OSA (495 ± pg/ml, p = 0.0113) **(D)** after six months of surgery.

A significant improvement in OSA indices, particularly in patients with moderate-to-severe OSA, was observed following surgical interventions (**Table 2**). We found a significant decrease in AHI six months after surgery (**Figures 5A, B**) in patients with none or mild and moderate-to-severe OSA (2.1 ±1.93 and 9.1 ±1.74 events/hour, p-value <0.0001). Additionally, our data demonstrated a significant decrease in plasma ANGPTL7 six months after surgery, which occurred in patients with none or mild and moderate-to-severe OSA (**Figures 5C, D**). Post-surgery analysis showed a wide improvement in lipid profile parameters, glucose metabolism indicators and inflammatory markers (**Table 2**). This involved a significant decrease in TG, cholesterol and LDL levels, while the improvement in other biomarkers such as oxLDL, IL-6 and IL-10 was not statistically significant.

**Table 2:** Relative levels of biomarkers before and after surgery in moderate-to-severe OSA.

	Before surgery	After surgery	P-value
	(N=31)	(N=31)
**BMI (kg/m^2^)**	43.7 ± 8.84	31.3 ± 6.77	<0.001
**c-peptide (pg/ml)**	5780 ± 2250	3570 ± 1820	<0.001
**Insulin (U/L)**	21.0 (9.8, 48.5)	5 (3, 8)	<0.001
**Glucose (mmol/L)**	6.7 (5.3, 8.4)	4.78 (4.47, 5.62)	<0.001
**AHI (events/h)**	28.7 ± 16.8	9.10 ± 9.69	<0.001
**AI (events/h)**	9.88 ± 12.0	4.19 ± 8.08	0.01
**HI (events/h)**	17.4 ± 9.29	4.92 ± 3.60	<0.001
**AHI-supine (events/h)**	33.5 ± 21.2	18.5 ± 19.2	<0.001
**CPAP (cm H_2_O)**	0.968 ± 0.180	0.806 ± 0.402	0.037
**Cholesterol (mmol/L)**	5.14 ± 0.879	4.30 ± 0.575	<0.001
**Triglyceride (mmol/L)**	2.30 ± 0.998	1.19 ± 0.418	<0.001
**HDL (mmol/L)**	1.24 ± 0.352	1.53 ± 0.441	<0.001
**LDL (mmol/L)**	3.27 ± 0.788	2.59 ± 0.509	<0.001
**ox-LDL (ng/ml)**	60.7 (35.4, 95.3)	59 (36, 91)	0.5
**ANGPTL7 (pg/ml)**	1,170.0 (322.5, 2,585.0)	495 (75, 1,075)	0.0113
**IL_6 (ng/ml)**	1.8 (0.8, 2.5)	1.36 (0.02, 1.90)	0.002
**IL_10 (ng/ml)**	2.1 (1.4, 2.5)	2.46 (1.33, 3.17)	0.019
**TNFα (ng/ml)**	2.6 (1.3, 4.1)	2.90 (1.20, 4.07)	0.5
**Leptin (pg/ml)**	51500 ± 42300	14300 ± 15400	<0.001

BMI, Body Mass index; AHI, Apnea Hypopnea Index; AI, Apnea Index; HI, Hypopnea index; HDL, High Density Lipoprotein; interleukin; LDL, Low Density Lipoprotein; ANGPTL7, Angiopoietin-like protein 7.

## Discussion

The current study investigated the correlation of ANGPTL7 and OSA severity. The results show a lower level of ANGPTL7 in patients with none or mild OSA while a significant elevation was shown in circulating ANGPTL7 and an increased expression of ANGPTL7 in subcutaneous white adipose tissue in patients with moderate-to-severe OSA. We further observed positive correlations between elevated ANGPTL7 expression and an increase in OSA severity. These findings highlight a potential role for ANGPTL7 in either OSA progression or in an OSA-related mechanism. Finally, amelioration of OSA with bariatric surgery concurred with a significant reduction in ANGPTL7 levels, which further emphasized the correlation between ANGPTL7 and OSA.

Bariatric surgery procedures have emerged as effective interventions for obesity and OSA. Although post-surgery improvement in OSA is well documented, biochemical factors involved in post-surgery OSA remission are still elusive. In this study, we found a correlation between ANGPTL7 and OSA indices at baseline. Also, we demonstrated a significant decrease in ANGPTL7 level that co-occurred with OSA remission post-surgery. ANGPTL7 is one of the least studied ANGPTL proteins, nonetheless it was found contributing to various pathways such as oxidative stress (17), inflammation (14), lipid metabolism (18), glucose metabolism and T2D (15). A previous study demonstrated that people with obesity had an increased expression of ANGPTL7. This increase was detected in adipose tissue and in the circulation, which was reverted by physical exercise (10). In this study elevated levels of ANGPTL7 in patients with obesity and OSA were found, which decreased after bariatric surgery. Levels of ANGPTL7 were significantly higher in patients with moderate-to-severe OSA compared to those with none or mild OSA, nonetheless levels of ANGPTL7 decreased significantly in both groups after the surgical intervention (**Figures 5C, D**). In addition, our data implicated a relation between ANGPTL7 and OSA which was supported by the significant positive correlation between ANGPTL7 and OSA indices. This was also emphasized by a multivariate logistic regression model which demonstrated that increased ANGPTL7 level coincided with an increase in OSA severity. Collectively, these findings highlight a potential role for ANGPTL7 in OSA progression, possibly through a direct or an indirect OSA-related factor.

**Figure 6:**
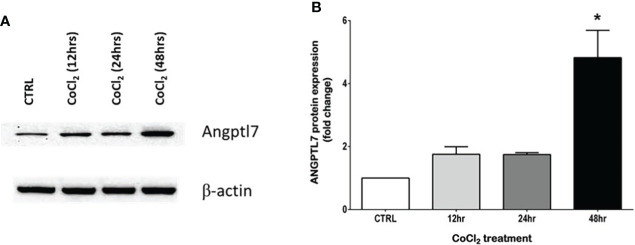
Hypoxic conditions increase ANGPTL7 expression. Effect of CoCl_2_ (150 µM), an eminent hypoxia imitative agent on ANGPTL7 expression in differentiated adipocytes (3T3-L1). **(A)** Whole cell lysates were separated on 8% SDS-PAGE and analyzed by Western blotting. Expression of ANGPTL7 showed an increase after 12hrs, 24hrs that was significant at 48hrs. **(B)** Protein quantification of ANGPTL7 that was normalized to β-actin showing statistical significance (p-value < 0.05) at 48hrs. Data represent mean ± SEM and it’s a representative of three independent measurements. *: p-value < 0.05.

ANGPTL proteins are secretory proteins that function as inhibitors of lipoprotein lipase (LPL) activity to hinder triglyceride-rich lipoproteins clearance and to consequently raise plasma concentration of TG (18). A number of ANGPTL family members, such as ANGPTL3, 4 and 8, play a prominent role in the regulation of TG metabolism (19). In a similar manner, ANGPTL7 appears to have a role in regulating TG metabolism. In agreement with the previously reported positive correlation between ANGPTL7 and TG in individuals with obesity (10), we find ANGPTL7 to have a significant positive correlation with TG in individuals diagnosed with OSA. The relation between ANGPTL7 and TG alludes to a role for ANGTPL7 in regulating TG metabolism in obesity, which was supported by the increase in ANGPTL7 expression after palmitate treatment (saturated fatty acid) in an *in vitro* setup (10). Recent evidence is suggesting a role for ANGPTLs in OSA. This notion has begun because lipid metabolism turned into an important frontier in OSA research after studies in animal models proved that chronic hypoxia results in dyslipidemia (20, 21). Additionally, a study demonstrated that chronic intermittent hypoxia could repress LPL activity by upregulating ANGPTL4 levels (22). Furthermore, Abu-Farha et al., reported elevated levels of ANGPTL4 and ANGPTL8 levels in patients with OSA (23). There is also new evidence suggesting that higher levels of ANGPTL3 in OSA patients can make them more prone to cardiovascular complications (24). In our study, the association data between the levels of ANGPTL7 and TGL prove the possible connection between lipid metabolism and OSA. Additionally, in an *in vitro* setup using differentiated adipocytes our data displays a significant increase in ANGPTL7 protein expression (**Figure 6**). This increase was induced after exposing the cells to Cobalt chloride (CoCl_2_), which is a hypoxia mimetic mediator that induces hypoxia-like responses. The rise in ANGPTL7 levels showed statistical significance after a prolonged exposure to hypoxic conditions, i.e. 48hrs (**Figure 6B**). However, more studies are warranted to decipher the exact mechanism of the connection and the role of ANGPTLs in regulating LPL activity in OSA.

In addition to lipid metabolism, ANGPTL7 also plays a role in oxidative stress, inflammation and cell adhesion through its role in TNF-α-induced endothelial dysfunction (17). An indicator of oxidative stress and lipid peroxidation is oxLDL, which showed a positive correlation with ANGPTL7 (**Figure 4C**). ANGPTL7 was suggested to reduce the production of nitric oxide by mitigating the catalytic effect of eNOS. Additionally, inhibition of ANGPTL7 significantly reversed TNF-α induced effect and it reduced cell adhesion of human umbilical vein epithelial cells (17). A main feature of OSA is intermittent hypoxia and the consequent systemic inflammation and endothelial damage (25). Intermittent hypoxia is associated with increased IL-6 levels, whereby patients with severe OSA had increased levels of IL-6 (4), which supports our current findings. Our data demonstrated increased IL-6 levels in patients with moderate-to-severe OSA, but the difference in IL-6 level was not significant compared to those with none or mild OSA. Roytblat et al. reported 34-fold increase in IL-6 levels in people with obesity, but an increase of 8-folds in patients with OSA compared to the control (26). This indicates that both obesity and OSA are factors contributing to the elevation of IL-6. However, obesity appears to have a stronger effect on IL-6 level. Since all of our study patients have obesity, this might explain the lack of significance in IL-6 level between our two study groups according to OSA severity. Moreover, the anti-inflammatory cytokine IL-10 that is involved in regulating tissue homeostasis (27) demonstrated a significant negative association with ANGPTL7, which supported the inflammatory role of ANGPTL7 in our cohort. Furthermore, levels of IL-6 showed a significant decrease after bariatric surgery while IL-10 exhibited a significant increase in patients with moderate-to-severe OSA. The changes in IL-10 and IL-6 levels following surgery coincided with the decrease in ANGPTL7 level which support the potential role of ANGPTL7 in inflammation.

One of the limitations in the current study is the lack of some of the polysomnography results such as oxyhemoglobin saturation, oxygen desaturation index, duration of apnea or hypopnea and the degree of sleepiness. Another limitation to our study is the fact that visceral adipose tissues were not collected. This could have given an insight to the possible differential expressions of ANGPTL7 between subcutaneous and visceral adipose tissue.

Although ANGPTL7 is presented as a promising biomarker with regard to its potential role in OSA, our current understanding of its physiological significance in OSA remains elusive. Our data support a correlation between ANGPTL7 and OSA severity. However, the functional significance of ANGPTL7 elevation is not clear. The finding that ANGPTL7 correlates positively with TG and oxLDL and negatively with HDL and IL-10 suggests a possible role for ANGPTL7 in oxidative stress, inflammation, and an increased CVD risk. Significant improvement in OSA has been demonstrated six months after bariatric intervention, which concurred with a significant decrease in circulating ANGPTL7. To the best of our knowledge, this is the first report to correlate increase in ANGPTL7 with an increase in OSA severity. Further studies are required to unveil the importance of ANGPTL7 in OSA, and its potential role as a biomarker in either the detection or management of OSA.

## Data availability statement

The original contributions presented in the study are included in the article/supplementary material. Further inquiries can be directed to the corresponding authors.

## Ethics statement

The studies involving human participants were reviewed and approved by OLVG Hospital, Amsterdam, the Netherlands. The patients/participants provided their written informed consent to participate in this study.

## Author contributions

ML, data analysis, initial manuscript drafting and revision. AA, conceptualization, study design and critical revision of the manuscript. MA-F, data analysis and interpretation and initial manuscript drafting and revision. PB, CR, CV, ES, data analysis and manuscript revision. TT and AC, data analysis and statistical analysis. IA-K and PC, performed BioPlex assay. SK, Confocal analyses. BW, Patient assessment and surgery. NV, conceptualization, study design and critical revision of the manuscript. JA, study design, data interpretation, wrote and critically revised the manuscript. All authors contributed to the article and approved the submitted version.

## Funding

This research was funded by Kuwait Foundation for the Advancement of Sciences (KFAS), for research project (RA 2016-023). The funders had no role in the design of the study, in the collection, analyses, or interpretation of data, in the writing of the manuscript, or in the decision to publish the results.

## Acknowledgments

We are grateful to Clinical Laboratory and the Tissue Bank Core Facility at DDI for their contribution in handling samples. We are also indebted to Kuwait Foundation for the Advancement of Sciences (KFAS) for financial support of this research project (RA 2016-023). The corresponding authors had full access to all the data in the study and had final responsibility for the decision to submit for publication.

## Conflict of interest

NV is a member of the Medical Advisory Board of NightBalance, consultant of Philips Healthcare, Inspire Medical Systems and Nyxoah.

The remaining authors declare that the research was conducted in the absence of any commercial or financial relationships that could be construed as a potential conflict of interest.

## Publisher’s note

All claims expressed in this article are solely those of the authors and do not necessarily represent those of their affiliated organizations, or those of the publisher, the editors and the reviewers. Any product that may be evaluated in this article, or claim that may be made by its manufacturer, is not guaranteed or endorsed by the publisher.
